# Periscope of Dermatology

**Published:** 1899-09-02

**Authors:** 


### Periscope of Dermatology.

**Eczema.**—Fox[1] advises in cases of eczema, accompanied by flatulent dyspepsia, that fluoride of ammonium should be given after each meal, and in cases where there is butyric fermentation the following cachet should be given with each meal: ℞. Erythyrol, 0·10 grmm.; calcined magnesia, 0·20 grmm. In the relapsing eczema of the gouty either the carbonate, the benzoate, or the salicylate of lithia may be given with advantage. With regard to infantile eczema it is pointed out that, although the sufferers appear to be in robust health, it will usually be found that there is either some disturbance of digestion, due to improper feeding, or that the breast-milk is unsuitable, or that there is failure of the balance between assimilation and excretion, usually due to overfeeding; these errors may, it is suggested, bring about an accumulation of toxic products in the blood. With regard to treatment, the causes of toxic infection must first be corrected, and the diseased surfaces must be protected from local sources of irritation; the parts should be bathed with bran or rice water, and a lotion, consisting of equal parts of black wash and lime water, with from 2ʒ to 10ʒ of mucilage of tragacanth to the pint, or the ordinary lotio calamine, should be applied. Prevention of scratching is best effected by applying splints to the extremities. Tar ointment may be employed at a later period.

Allen[2] recommends, in a case of infantile eczema, that (1) the clothing, especially the diaper, should be inspected; (2) the bath and meals should be regulated; (3) the mother's milk should be examined both as to quality and quantity; (4) intestinal derangements of both mother and child should be corrected; and (5) the skin should be protected from irritation caused by improper soaps, water, the sun, and cold winds, &c. In cases of eczema below the scalp the head must be kept free from the dirty or greasy crusts so often present. For this purpose he uses the following prescription: ℞. Resorcin, 5; washed sulphur, 2; lanolin, 5 parts in 100 of vaseline. In eczema about the ano-genital region he applies a 3 per cent. solution of methylene blue, its use being analgesic, protective, and antiseptic.

Brault[3] reports a case of eczema seborrhoica, in which the eruption disappeared after injections of calomel. The patient was a man, aged 40, who had previously suffered from syphilis, but who had typical seborrhoic eczema on the chest, the centre of the plaques being covered with greasy scales. The lesions faded and disappeared after two injections of calomel, but returned after an interval of about six weeks.

Radaeli[4] reports the results of a trial of picric acid in different forms of eczema. The affected parts were first freed from scabs, &c., the hair cut as short as possible, and the whole region thoroughly washed with boracic acid solution. The part was dried, and a saturated watery solution of picric acid was painted on, and a compress soaked in the same solution was applied, over which a layer of cotton wool was placed to absorb the discharge. The dressing was left on for one or two days. The remedy has the disadvantage that it causes great smarting in the parts to which it is applied; this, however, ceases completely in about 15 minutes, giving place to a sense of relief, which is mainly due to the cessation of itching. The dressing is especially convenient in cases of weeping eczema, in which there is much discharge.

**Lupus.**—Bernstein[5] has collected the reported cases of lupus treated by calomel injections. In 37 cases there were positive results, and in 10 negative. The dose of each injection was 1 c.cm. of emulsion of calomel in olive oil (1 in 10). Six injections were given within eight days. Deutsch also records a case in which injections of grey oil were used for a secondary syphilide in a patient who was also afflicted with lupus. Under this treatment the lupus showed considerable improvement.

Krzysztalowiccz[6] reports 13 cases treated with Koch's new tuberculin; 11 were cases of lupus, one had tuberculosis of the skin and bones, and another tuberculous ulceration of the genitals. The amount injected varied from 1-500th mgrm. to 20 mgrms., and the number of injections from 18 to 51. Constitutional reaction was slight, and local reaction only occurred after large doses. The author concludes that lupus improves, but is not cured, by this treatment.

**Therapeutics.**—Max Möllers[7] reports the results of the employment of endermol in scabies. Of 67 cases treated by friction with a 1 per cent. ointment of endermol, 64 were cured after one application, the other three requiring six applications. The ointment should be left on for two days, the patient then taking a bath and changing his underclothing. The drug is the salicylate of nicotine, and causes neither irritation nor toxic symptoms.

**Airol.**—Speigel[8] reports the unpleasant consequences which may follow the use of airol. Pain and bullous lesions followed its application to a sore; great pain and iodism followed the injection of a 10 per cent. solution into an abscess; vesicular bullous dermatitis, involving the back of the hand and the fore-arm, followed its application to a sub-ungual whitlow. The irritation was allayed in each case by the use of equal parts of lime water and powdered talc.

**Antipyrin.**—Wechselmann[9] gives a description of the varieties of skin eruptions met with after the use of antipyrin, and refers to five cases under his own observation—(1) A man, aged 40, who had been in the habit of taking the drug freely for migraine, suddenly developed a vesicular eruption about his mouth, and front part of the tongue; the penis, scrotum and anus being similarly attacked. On discontinuing the drug the eruption disappeared, but reappeared when a small dose was again taken. (2) A woman, aged 40, took antipyrin for migraine; the lips, eyelids, tongue, and dorsal aspects of both hands became the seat of a painful vesicular eruption. This also recurred after taking another dose of the drug. (3) A man, aged 62, suffering from diabetes, after taking antipyrin for some time, noticed a hæmorrhagic eruption on the dorsal surfaces of the left hand, the skin being œdematous. This also disappeared immediately the drug was discontinued. (4) A diabetic man, aged 66, for six years had suffered from a vesicular eruption on the dorsal surfaces of both hands, the lower lip, the anus, and scrotum; this occurred every second year. (5) A man, aged 29, who had suffered from periodic attacks of so-called eczema, developed a similar eruption shortly after taking three grains of antipyrin. Vesicles formed in the perineum and on the dorsal surface of the hands and toes. He had no more attacks when he ceased to take antipyrin.

[1] Clin. Jour., May 10, 1899, p. 48. [2] New York Med. Jour., April 1, 1899; Inter. Med. Mag., May, 1899, p. 371. [3] New York Med. Jour., April 1, 1899, p. 370; Ann. de Derm. et de Syph., Jan., 1899. [4] Brit. Med. Jour. Epit., Mar. 11, 1899; La Semaine Med., Feb. 18, 1899. [5] Brit. Med. Jour. Epit., Mar. 11, 1899; Munch. Med. Woch., Nov. 15, 1898. [6] Wien, Med. Woch., No. 2, 1899; Brit. Med. Jour. Epit., Mar. 11, 1899. [7] Rev. Inter. de Ther. et de Pharm., 1899, p. 331; Postgraduate, Mar., 1899, p. 245. [8] Monatsh. f. Prakt. Derm., 27, No. 12, 1899, p. 628; Brit. Jour. of Derm., June, 1899, p. 260. [9] Brit. Med. Jour. Epit., Jan. 14, 1899; Deut. Med. Woch., May 29, 1899.

